# Pediatric Chylothorax Treated With Lymphangiography and Thoracic Duct Embolization: A Case Report

**DOI:** 10.7759/cureus.63981

**Published:** 2024-07-06

**Authors:** Harneet Sangha, Nathan G Savolainen, Douglas A Murrey, Jason S Vergnani

**Affiliations:** 1 Interventional Radiology, Elson S. Floyd College of Medicine, Spokane, USA; 2 Interventional Radiology, Inland Imaging, Spokane, USA

**Keywords:** thoracic duct, interventional radiology, chylothorax, thoracic duct embolization, lymphangiogram

## Abstract

A chylothorax, the accumulation of lymphatic fluid in the pleural space, may occur for a variety of reasons. It is commonly seen in adults post-thoracic surgery. We present the case of a seven-month-old girl with a right-sided chylothorax in the setting of non-accidental trauma. Treatment options for a chylothorax include surgical ligation of the thoracic duct or, as in this case, a minimally invasive procedure performed by interventional radiology known as lymphangiography with thoracic duct embolization. This case highlights interventional radiologists' ability to treat complex lymphatic pathologies effectively with minimally invasive techniques.

## Introduction

Chylous leaks are rare occurrences that happen when intestinal lymph, also known as chyle, escapes from the lymphatic system. They can happen at any point along the path that the chyle follows, starting from the lymphatic ducts in the intestines, passing through the cisterna chyli, and extending into the thoracic duct. These leaks lead to various clinical manifestations, including chylothoraces, chylopericardium, chylous ascites, etc. 

The majority of chyle leaks stem from medical procedures, particularly surgeries on the neck, chest, or abdomen, where damage to the thoracic duct (TD) or its branches can occur. However, these leaks can also arise due to obstructions or trauma [1]. Non-traumatic chylous leaks are uncommon occurrences, often attributed to conditions that lead to the blockage of lymphatic vessels. These conditions may include malignancies like lymphoma, diseases affecting lymphatic vessels such as Gorham's disease, systemic disorders like sarcoidosis or Behcet's disease, congenital malformations, and cases with no known cause (idiopathic) [2]. In a recent case series by Nadolski and Itkin, it was found that idiopathic causes accounted for 10-42% (14/34) of non-traumatic chylothorax cases [3,4]. 

Lymphangiography (LG) is a valuable diagnostic tool for identifying various types of lymphatic leaks, including chylothoraces, chylous ascites, and lymphatic fistulae [5,6]. Previous research has indicated detection rates for leaks ranging from 64% to 78% [5-7]. In addition to its diagnostic function in detecting lymphatic leaks, recent publications suggest that LG also serves a therapeutic purpose of managing lymphatic leakage [5,8]. The precise mechanism by which LG mitigates lymphatic leaks remains insufficiently understood, although some researchers propose that Lipiodol, an ethiodized oil contrast agent used in LG, triggers an inflammatory and granulomatous response upon extravasation [5].

Once the site of the leakage or blockage has been identified via LG, a procedure called thoracic duct embolization (TDE) is performed. TDE is a minimally invasive procedure guided by imaging techniques to occlude the thoracic duct (TD). Developed by Dr. Cope, a prominent early interventional radiologist, this technique initially aimed to provide an alternative to surgical ligation of the TD [9,10]. It involves a three-step process: first, LG is performed, followed by percutaneous catheterization of the cisterna chyli (CC) through the abdomen, and finally, embolization of the TD above the site of leakage or obstruction; the technical success rates of TDE have been reported to be around 70% in the literature [11].

We herein report a case in which TDE and LG were used to treat a chylothorax in the setting of non-accidental/trauma in a seven-month-old infant. This case aims to highlight the effectiveness of interventional radiology (IR)-based techniques for chylous effusions.

## Case presentation

A seven-month-old infant presented to the Emergency Department (ED) with respiratory distress, poor perfusion, metabolic acidosis, and hepatomegaly. The patient was found to have a pleural effusion and was treated with a chest tube and weaning high-flow nasal cannula, leading to improvement in symptoms in the acute ED setting. After the examination of the pleural fluid, the color and consistency were described as strawberry milk with 90% lymphocytes, which is consistent with the diagnosis of a chylothorax. Additionally, skin lesions and suspicious lesions on a chest X-ray indicated non-accidental trauma, and child protective services were notified. 

**Figure 1:**
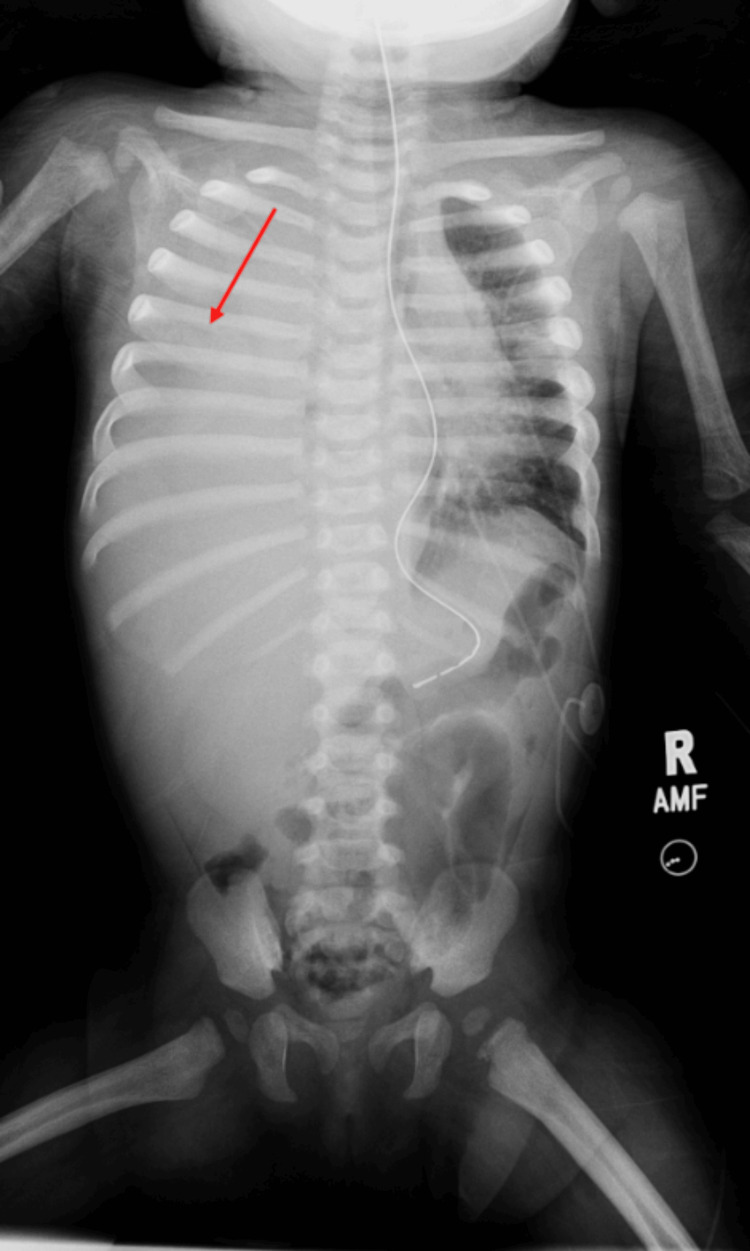
Chest abdomen radiograph showing near-complete opacification of the right hemithorax concerning for a large pleural effusion in addition to a periosteal reaction of the left ribs and the proximal right humerus suspicious for subacute fractures and concerning for non-accidental trauma.

**Figure 2:**
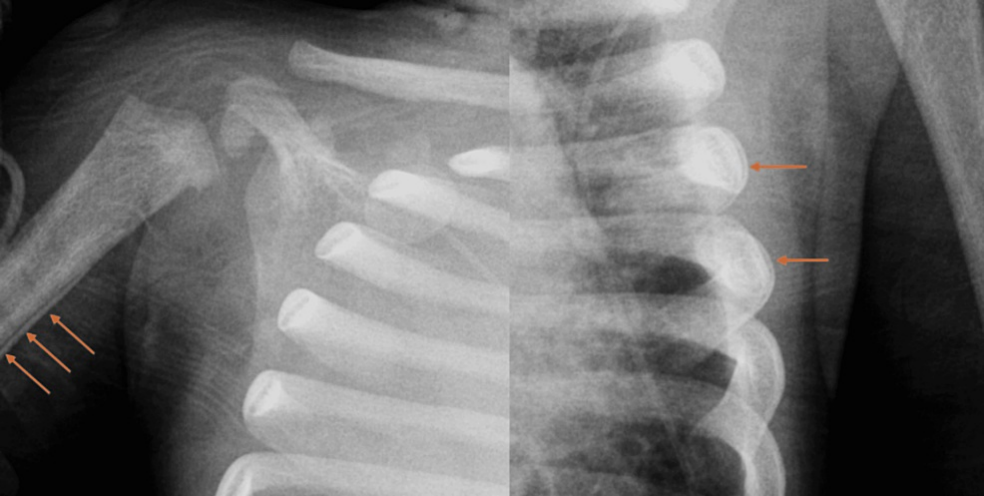
Close-up of the periosteal reaction.

The chest X-ray findings indicated a complete opacification of the right hemithorax (chylothorax was confirmed with elevated triglycerides in the fluid) and possible areas of asymmetric periosteal thickening involving partially imaged right humerus and at least two left ribs (Figures 1, 2). Axial CT with IV contrast showed the right chest tube and a periosteal reaction of the left ribs suspicious for non-accidental trauma. The patient was started on total parenteral nutrition (TPN) and then nothing by mouth (NPO). This led to the worsening of the chylothorax and IR was consulted for LG with embolization.

**Figure 3:**
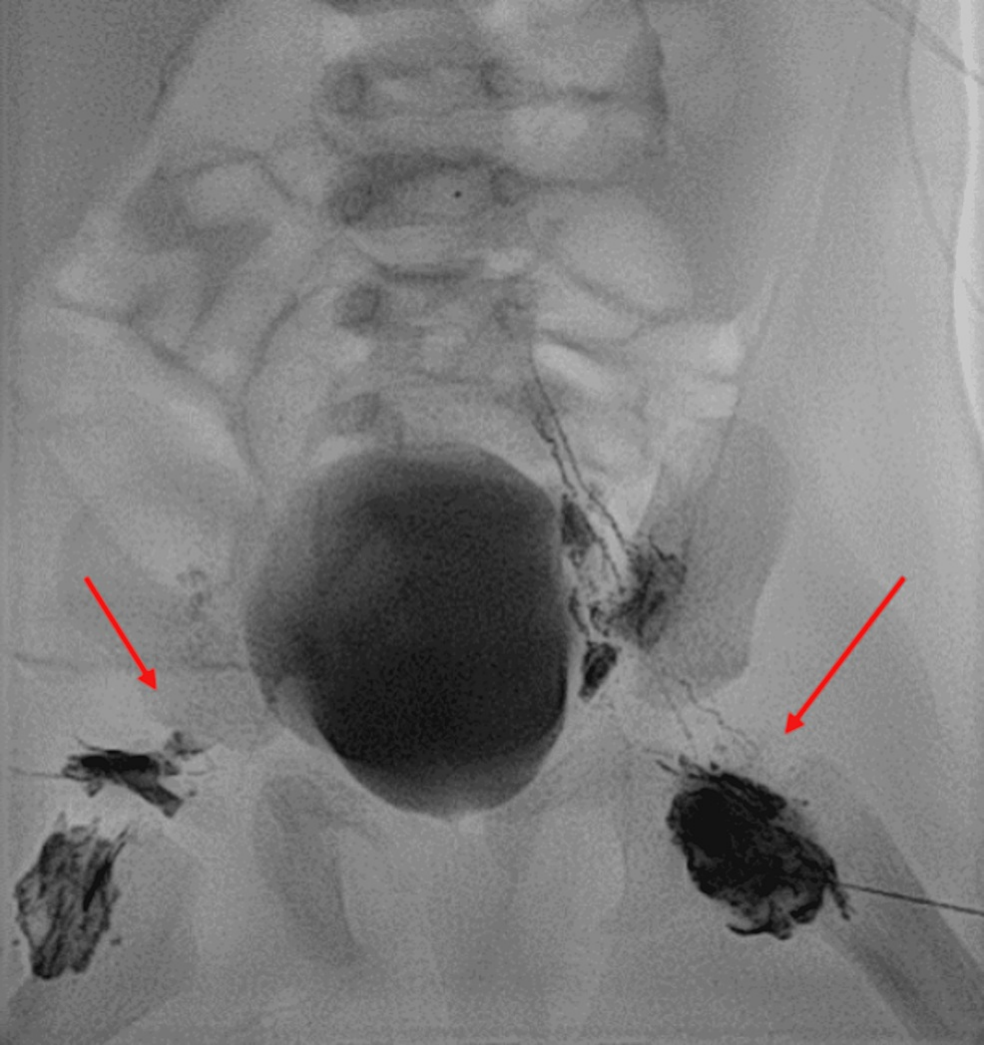
Lymphangiogram showing the injection of Lipiodol into bilateral inguinal lymph nodes with antegrade flow through pelvic lymphatic channels.

**Figure 4:**
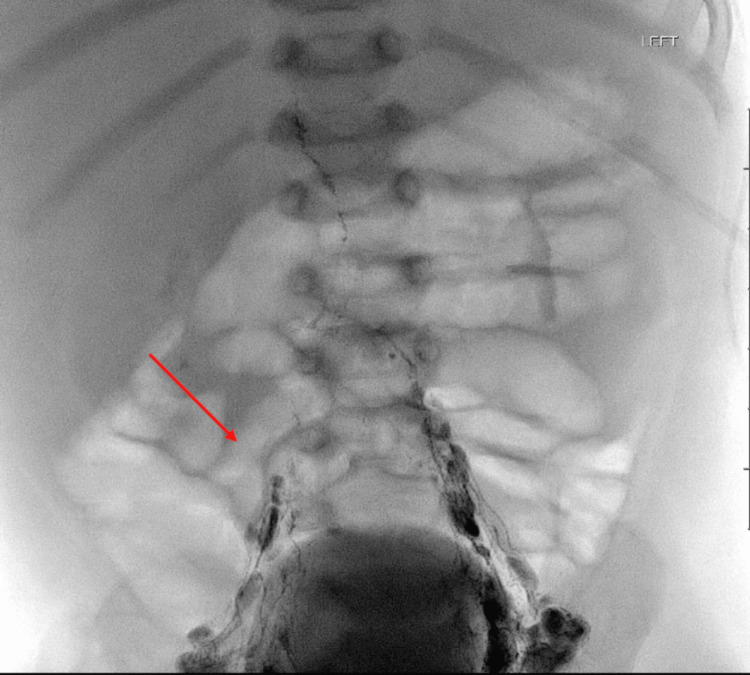
Fluoroscopic image showing the continued flow of Lipiodol, which is now accumulating in the cisterna chyli near the right aspect of the T12 vertebral body.

**Figure 5:**
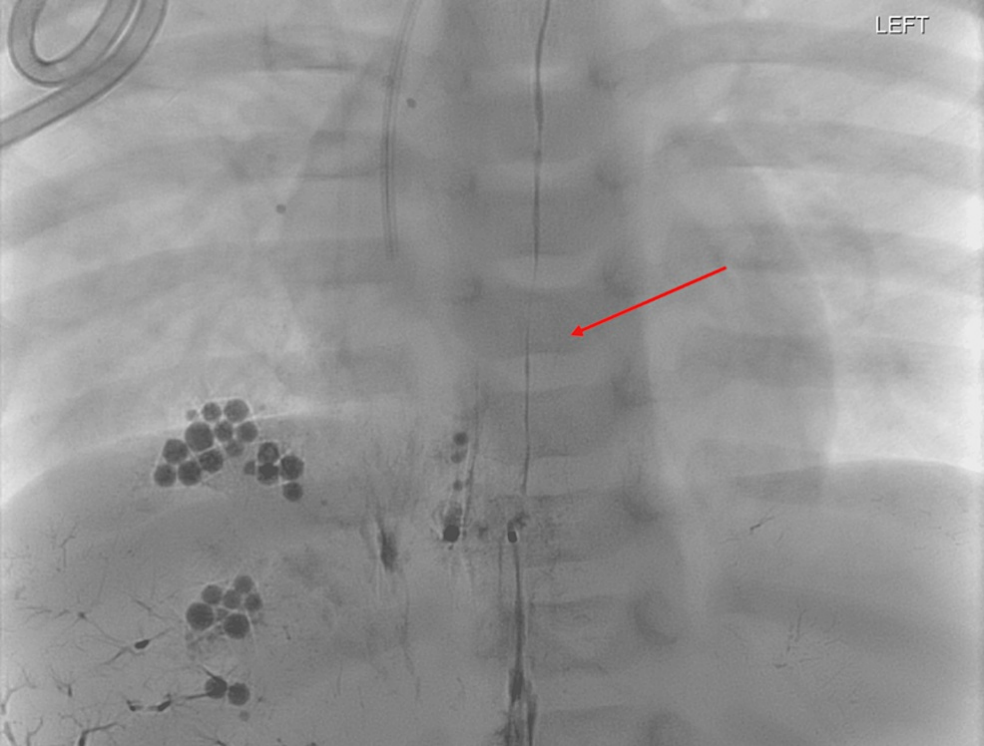
Fluoroscopic spot image showing liquid embolic throughout the thoracic duct in addition to the accumulation of Lipiodol in the dependent right pleural space.

**Figure 6:**
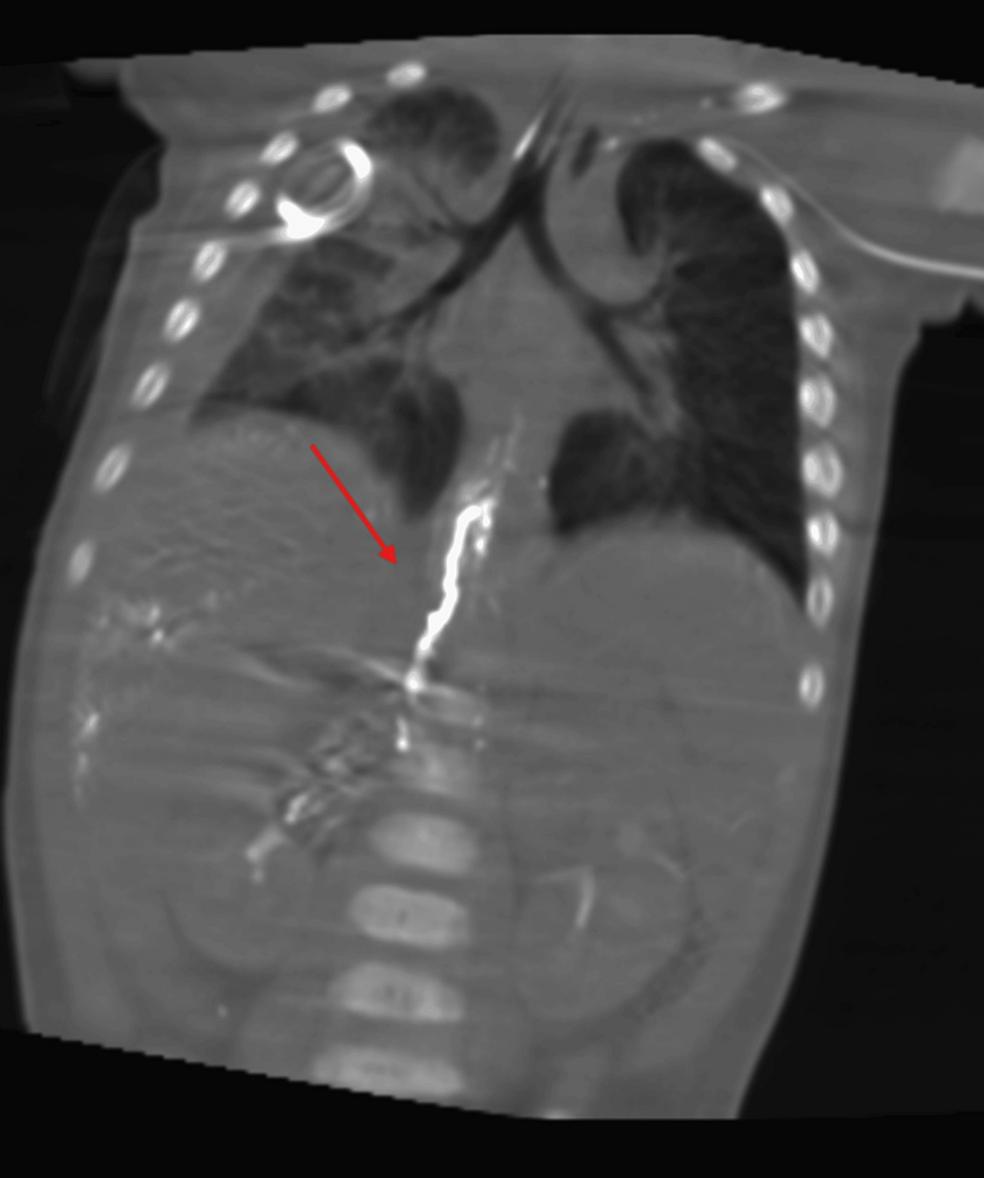
Coronal reformat of cone beam CT showing liquid embolic in the thoracic duct.

IR treated the patient with LG and TDE on March 26 (Figures 3, 4). Obsidio liquid embolic was used for the occlusion of the cisterna chyli of the abdominal thoracic duct (Figures 5, 6). The chest tube was removed on April 1 and the repeat X-ray was stable without reaccumulating fluid. The patient underwent a swallow study, which was severely abnormal with almost complete aspiration leading to NPO with TPN. A nasogastric tube was placed, and she was transitioned to low-fat tube feeds for the remainder of her admission. The patient tolerated this well and was gaining weight prior to discharge. She was referred to a speech and feeding clinic for ongoing speech therapy with a hopeful transition to oral feeds eventually. She has an outpatient dietitian to transition to full-fat feeds after four weeks on Monogen (Nutricia Metabolics, Utrecht, The Netherlands).

## Discussion

A chylothorax is a rare condition where lymphatic fluid accumulates in the pleural space [12]. Chyle production is around 2.4 L per day, which means a considerable amount can accumulate in the pleural space [13]. Chyle can accumulate for two days to several weeks before the patient becomes symptomatic with pleural effusion symptoms. If left untreated, patients with recurrent or large chylothorax may develop hypovolemia, malnutrition, vitamin deficiencies, electrolyte imbalances, and immunosuppression [14]. Chyle is described as having a milky white appearance; however, this color is described in less than half of the patients with chylous effusions [12].

The reasons for the development of a chylothorax can be grouped into three categories: traumatic, spontaneous (non-traumatic), and idiopathic reasons. Some reasons for traumatic chylothorax include postoperative, with esophagostomy being the highest risk, blunt trauma to the chest or thoracic spine, and penetrating injuries [13]. Some non-traumatic causes include malignancies, the most common being lymphomas, idiopathic partial or complete obstruction of the TD, congestive heart failure, and cirrhosis [12,14]. Idiopathic causes make up about 10% of all chylothorax patients [4]. 

This case highlights the use of IR in non-accidental trauma/abuse to effectively treat a chylothorax using minimally invasive techniques. After this patient underwent LG with TDE, the chylous effusion was essentially cured with the removal of the chest tube. Tamura et al. found a similar result in their case study of a cervical chylous leakage that was treated successfully with Lipiodol LG [15]. In Wagenpfeil et al.'s results, 15/17 (88.2%) of effusion cases were resolved, with 10/12 (83.3%) resolved after angiography alone and 5/5 (100%) after embolization in non-traumatic chylous effusions. They went on to say that the lymphatic abnormalities in a majority of the patients after LG reside and lead to resolution in >80% of cases [16]. When discussing lymph vessel embolization, the success rate is well over 90% of patients when the TD is intubated successfully [5].

Non-accidental trauma and the radiologist’s role in recognizing and informing care should also be brought to attention. Fractures are the second most common finding in child abuse after skin lesions such as bruises [17]. While they are the second most common cause, they are hard to identify and diagnose [17]. The help of a pediatric radiologist should be consulted in these cases because abuse is substantially underdiagnosed compared to overdiagnosis [17,18]. Brown argued that child abuse physicians, including pediatric radiologists, should build trust with criminal defense advocates by acknowledging the harms associated with false positives and false negatives [18]. This would help alleviate the unhealthy circumstances while physicians are trying to keep the child's best interest in mind [18]. 

The IR-based treatment of a chylothorax using LG and subsequent embolism has a minimally invasive procedure and effective outcomes [11,15,16]. Visualized leak of the chylous on LG and embolism of the TD showed improvement in this patient with no reaccumulating fluid; however, there are limitations to this study. This is a single case study and more investigations should be done on the embolization safety and patient benefit. Moreover, there is TD anatomic variation in 40-60% of patients, and tributaries to the main thoracic duct are common [14]. This could become an issue in some patients when trying to embolize the TD and cause recurrent chylothorax. 

This study identifies a patient who improved from an identified chylothorax on LG with embolism. The patient benefited from no chylous fluid reaccumulating. 

## Conclusions

This case report demonstrates the effective use of interventional radiology procedures, specifically lymphangiography and thoracic duct embolization, in treating a complex chylothorax in a seven-month-old infant resulting from non-accidental trauma. The minimally invasive nature of the procedure facilitated a successful outcome with no recurrence. The role of interventional radiologists is crucial not only in the treatment but also in the accurate diagnosis of non-accidental trauma cases. 
